# Supplementary material for: Cyclic thermal stress during pupation triggers irreversible carry-over effects in a key pollinator
Source: Commun Biol. 2026 Sep 25;9:1228. doi: 10.1038/s42003-026-10940-3 (PMC13614927; doi:10.1038/s42003-026-10940-3)
Supplement: Supplementary file 1 [file 42003_2026_10940_MOESM1_ESM.pdf]

Supplementary Material

Cyclic thermal stress during pupation triggers irreversible carry-over effects in a key pollinator

Maximilian M. Mandlinger[1] and Christoph Kurze[1]*

[1] Evolutionary Ecology, University of Regensburg, Germany
* Corresponding author, email: Christoph.Kurze@ur.de

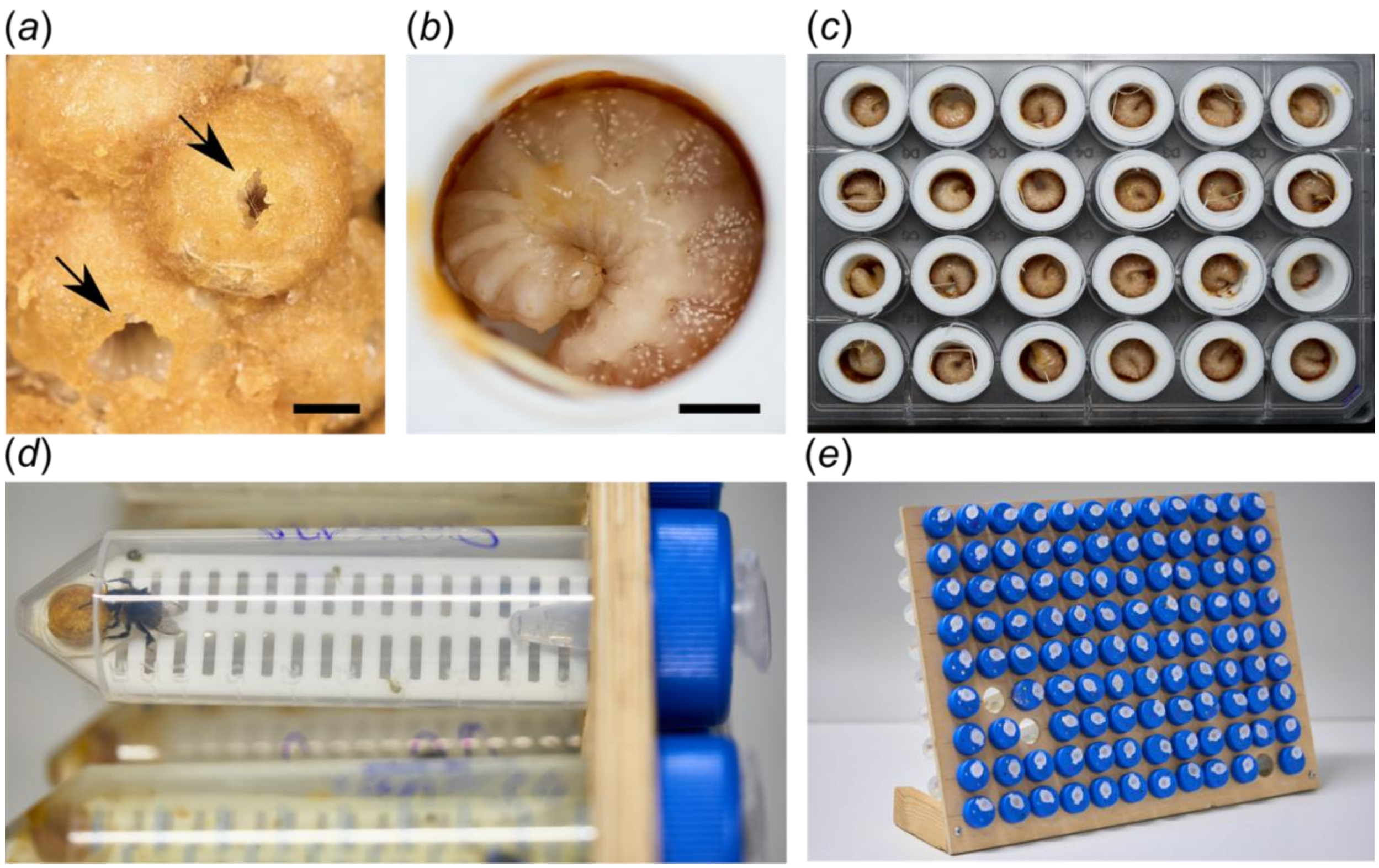

Supplementary Figure 1. Experimental setup for *in vitro* rearing and adult longevity assay in *B. terrestris*. (*a*) Fourth-instar larvae (L4) were identified by the characteristic small feeding holes (black arrows), collected, and transferred into (*b*) 3D-printed polylactide (PLA) artificial brood cells for *in vitro* rearing (see methods). These cells were within (*c*) a 24-well plate setup inside humidity and temperature controlled incubators. (*d*) Newly emerged adults were individually housed in modified 50 mL tubes, serving as individual cages. Each caged contained a 3D-printed PLA mesh insert for sanitary reasons, a 1.5 mL perforated tube providing *ad libitum* 60% w/v sucrose solution, and pollen candy. (e) Individual cages were randomly stored in a wooden rack within the insectary room of their natal colonies, maintained at standardized conditions (25 ± 1°C and 30-50% RH). Black scale bars = 2.5 mm.

(*a*)

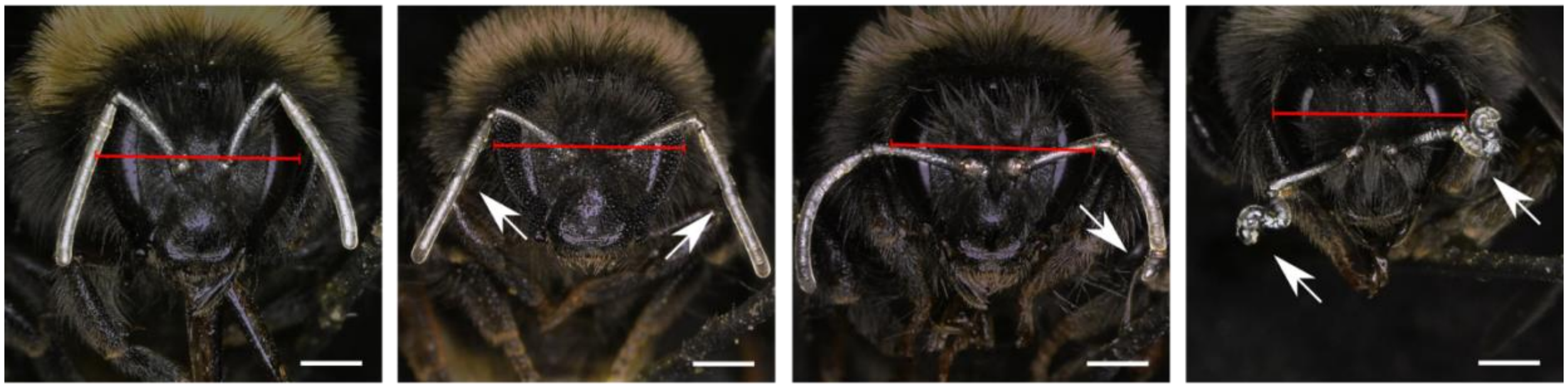

(*b*)

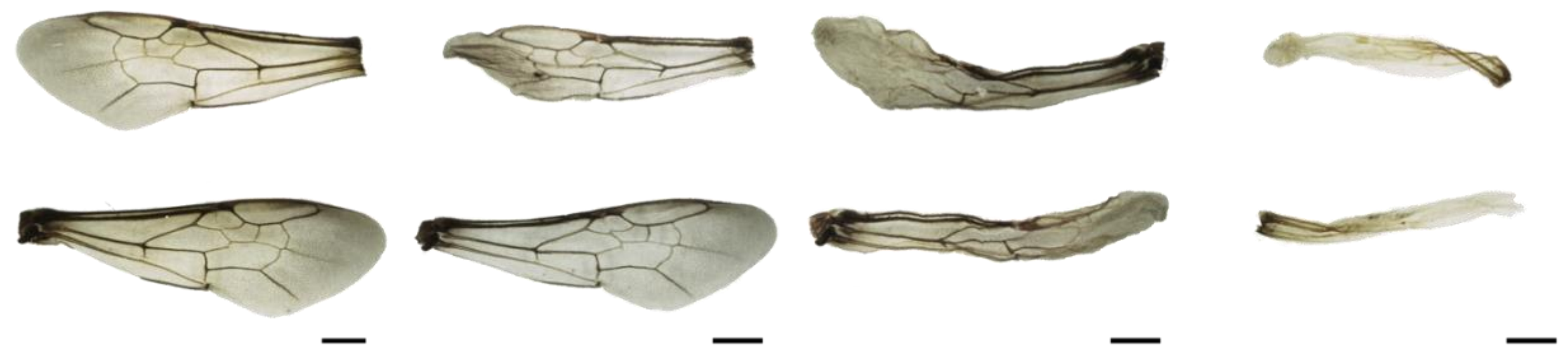

Supplementary Figure 2. Representative examples of morphological deformations in adult *B. terrestris*. (*a*) Examples ranging from an individual with normally developed antennae (left) to increasingly severe deformations (right). White arrows indicate regions of deformations, including slightly deformed, fused, missing and abnormally curved flagellomeres. Red lines indicate head width measurements. (*b*) Paired left and right forewings from an adult bee with normal development (left) to examples with increasingly more severe deformations (right). These images serve as representative examples of what we scored as deformations in our analyses (see methods; Fig. 2*e* and 3*a*). For the presentation purposes, photographs were enhanced by colour corrections (heads and wings), shading (heads), removing backgrounds, and aligning (wings) using graphical software. Scale bars = 1mm.

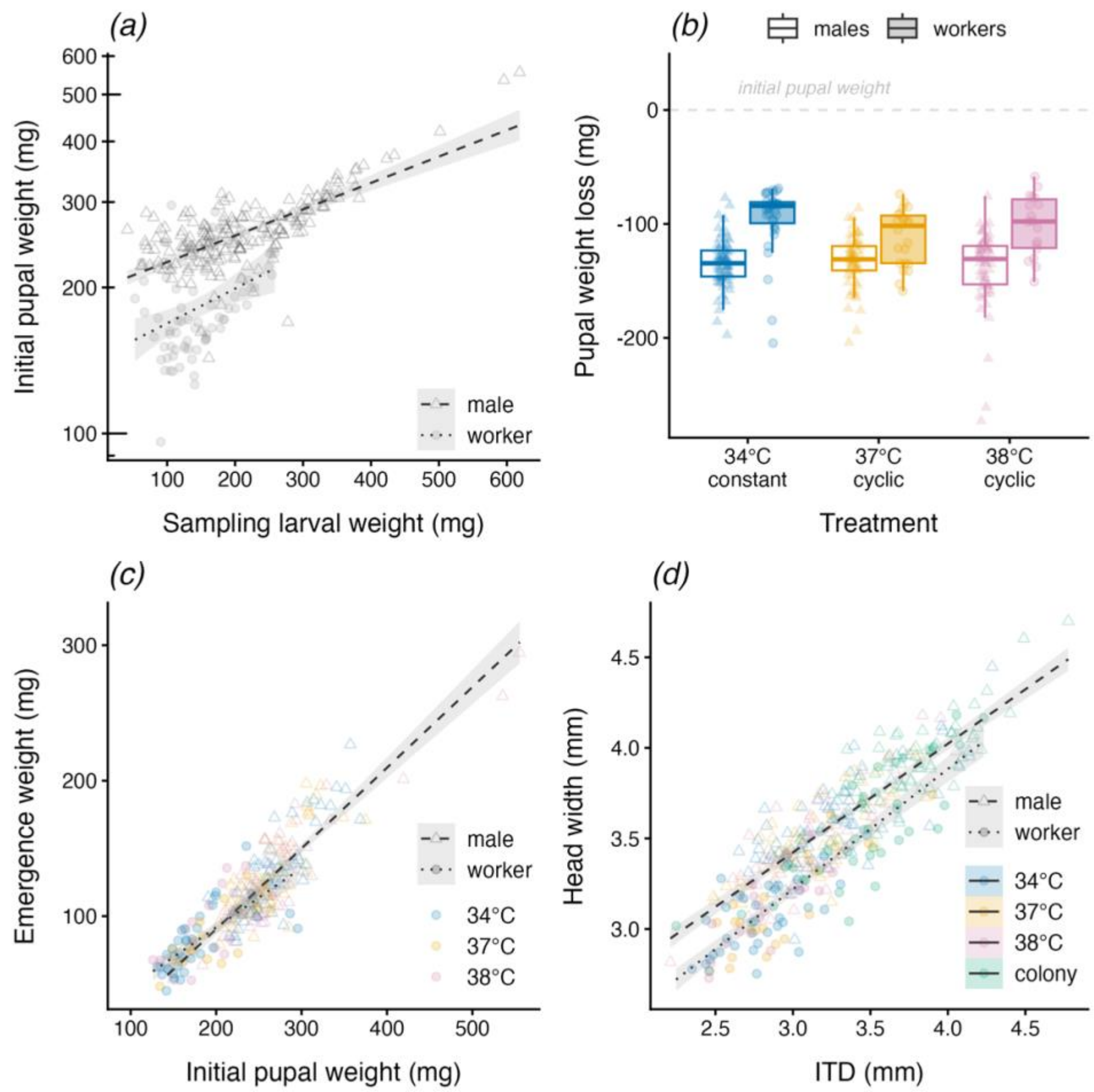

Supplementary Figure 3. Morphometric relationships from initial sampling of L4 larvae to adulthood in *B. terrestris* males and workers. ***(a)*** There was a strongly positive relationship between the sampling weight (mg) of L4 larvae from their natal colonies and their initial pupal weight (on log10 scale) at the beginning of the experiment (lognormal GLMM: $\chi^2 = 260.65$, $p < 0.0001$), with pupal weight differing between sexes ($\chi^2 = 110.82$, $p < 0.001$). ***(b)*** Box plots show the median (centre lines) absolute weight loss during pupation (initial pupal weight – weight at emergence) for each *in vitro* rearing treatment and sex (open boxes = males; filled boxes = workers). The bottom and top of the boxes represent the 1st and 3rd quartiles, respectively. The proportional weight loss (relative to initial weights) and statistical analysis are presented in the main manuscript (see Fig. 1*d*). ***(c)*** There was strong positive linear relationship between initial pupal weight (mg) and emergence weight (mg) (GLMM: $\chi^2 = 402.03$, $p < 0.0001$; Pearson's correlation: $r = 0.88$), which was independent of treatment (blue = constant 34°C; orange = cyclic 37°C; reddish-purple = cyclic 38°C), but a marginally sex-dependent effect ($\chi^2 = 4.03$, $p = 0.045$). ***(d)*** Morphometric relationship between ITD (inter tegular distance) and head width measured in adults of colony-reared (green = colony) and *in vitro*-reared individuals (i.e. experimental treatments: blue = constant 34°C; orange = cyclic 37°C; reddish-purple = cyclic 38°C). ITD was a strong predictor of head width (GLMM: $\chi^2 = 419.53$, $p < 0.0001$; Pearson's correlation: $r = 0.87$), with indistinguishable scaling relationships between sexes (both slopes = 0.353), but with males being larger than workers ($\chi^2 = 5.43$, $p < 0.05$). ***(a, c, d)*** Dashed (males) and dotted (workers) lines show fitted trends ± 95% CI error bands (grey) from GLMs. ***(a-d)*** Individual data points in all plots are displayed as triangles (males) and circles (workers). Sex-specific samples sizes for 34°C ($n_m = 66$, $n_w = 35$), 37°C cyclic ($n_m = 58$, $n_w = 24$), and 38°C cyclic ($n_m = 60$, $n_w = 18$).

Table S1. Pairwise comparisons of slopes describing the scaling relationship between emergence weight and head width across treatments and sexes. Sloped (estimated marginal trends) were obtained from a GLMM that included a significant three-way interaction between log(emergence weight), treatment (34 °C constant, 37 °C cyclic, 38 °C cyclic, and colony-reared), and sex (males and workers). Pairwise comparisons of slopes were conducted using two-sided tests with Holm-Bonferroni correction. Despite this interaction, scaling relationships remained largely consistent across all groups (see Fig. 3c).

Pairwise comparisons	Estimate	SE	df	z ratio	p value
34°C male – 37°C male	0.0193	0.0239	Inf	0.8105	1.0000
34°C male – 38°C male	-0.0088	0.0215	Inf	-0.4122	1.0000
34°C male – colony male	0.0442	0.0202	Inf	2.1819	0.6406
34°C male – 34°C worker	0.0440	0.0256	Inf	1.7202	1.0000
34°C male – 37°C worker	-0.0210	0.0300	Inf	-0.6985	1.0000
34°C male – 38°C worker	0.0829	0.0304	Inf	2.7266	0.1664
34°C male – colony worker	0.0122	0.0219	Inf	0.5571	1.0000
37°C male – 38°C male	-0.0282	0.0235	Inf	-1.1969	1.0000
37°C male – colony male	0.0248	0.0225	Inf	1.1059	1.0000
37°C male – 34°C worker	0.0246	0.0272	Inf	0.9054	1.0000
37°C male – 37°C worker	-0.0403	0.0313	Inf	-1.2868	1.0000
37°C male – 38°C worker	0.0636	0.0319	Inf	1.9898	0.9150
37°C male – colony worker	-0.0071	0.0239	Inf	-0.2986	1.0000
38°C male – colony male	0.0530	0.0200	Inf	2.6534	0.1992
38°C male – 34°C worker	0.0528	0.0252	Inf	2.0955	0.7586
38°C male – 37°C worker	-0.0121	0.0297	Inf	-0.4085	1.0000
38°C male – 38°C worker	0.0918	0.0302	Inf	3.0405	0.0661
38°C male – colony worker	0.0210	0.0216	Inf	0.9762	1.0000
colony male – 34°C worker	-0.0002	0.0242	Inf	-0.0081	1.0000
colony male – 37°C worker	-0.0651	0.0290	Inf	-2.2470	0.5667
colony male – 38°C worker	0.0387	0.0294	Inf	1.3185	1.0000
colony male – colony worker	-0.0320	0.0203	Inf	-1.5755	1.0000
34°C worker – 37°C worker	-0.0650	0.0325	Inf	-1.9977	0.9150
34°C worker – 38°C worker	0.0389	0.0332	Inf	1.1718	1.0000
34°C worker – colony worker	-0.0318	0.0256	Inf	-1.2420	1.0000
37°C worker – 38°C worker	0.1039	0.0368	Inf	2.8257	0.1274
37°C worker – colony worker	0.0332	0.0301	Inf	1.1023	1.0000
38°C worker – colony worker	-0.0707	0.0305	Inf	-2.3213	0.4864
