# Supplementary material for: Cyclic thermal stress during pupation triggers irreversible carry-over effects in a key pollinator
Source: Commun Biol. 2026 Sep 25;9:1228. doi: 10.1038/s42003-026-10940-3 (PMC13614927; doi:10.1038/s42003-026-10940-3)
Supplement: Supplementary file 2 [file 42003_2026_10940_MOESM2_ESM.pdf]

nature portfolio

Peer Review File

Cyclic thermal stress during pupation triggers irreversible carry-over effects in a key pollinator

Corresponding Author: Dr Christoph Kurze

This file contains all reviewer reports in order by version, followed by all author rebuttals in order by version.

Version 0:

Reviewer comments:

Reviewer #1

(Remarks to the Author)
This manuscript demonstrates both lethal and sub-lethal effects resulting from thermal stress experienced during development. This study is well done and addresses a gap in the understanding of how global climate change impacts bumblebees by looking at how temperatures experienced during development impact adults. This manuscript was very well written, and I do not have many edits. The experiments were well-designed, and the figures represent the results well. I have included some of my recommendations to improve the manuscript below.

Introduction:

Lines 33-34: include examples of sublethal effects that have previously been found

Lines 48-50: include information about how adults regulate temperature and how the larvae/pupae experience different temperatures than the adults.

Line 58: Does “a reduction in adult emergence” mean decreased survival? This phrasing is confusing

Line 58: change “ab” to “an”

Line 64-65: Change the phrasing of “carry-over risk” to something like residual risk, cumulative risk, or carry-over effects

Methods:

Lines 108-111: How long are the bees exposed to the temperature treatments? Did they experience 1 round of the cyclic thermal regime? Or throughout development? If so, how long were the bees exposed (in days). Please clarify.

Line 137: change “contacted” to “connected”

Lines 137-141: clarify that you are working with adult bees. Also, it is to be assumed that these are adult bees that emerged from the pupal treatments? If that is the case please clarify here.

Lines 146-148: State here briefly why you measured dry mass twice. It is stated in the following paragraph, but a brief statement would help clarify when first mentioned.

Line 149-150: include citations for the extraction method

Line 159: change “re-drying” to “re-dried”

Results:

Line 209: report actual p value

Line 213: Again, change the term “carry-over” for clarity, and throughout the manuscript

Line 214: does “adult survival” refer to survival to adulthood? Or is it talking about adults exposed to temperature treatments. Please clarify.

Fig. 1a change the title of the y-axis to reflect that it is a proportion

Line 264: include actual pvalue

Lines 273-274: did you account for body size within relative lipid content? If so please describe how this was accomplished in your analysis.

Discussion:

Lines 314-327: Deformities due to temperature stress has also been seen in the solitary bee Megachile rotundata, it might be helpful to include this reference to further explain your findings. doi:10.1242/jeb.243242. note this article is about low temperature stress.

Reviewer #2

(Remarks to the Author)
To the authors:
The authors of “Deferred mortality: cyclic thermal stress during pupation triggers irreversible carry-over costs in a key pollinator” investigated how fluctuating thermal stress during development affects bumblebee physiology including developmental time, survival, and morphology. The study, in my opinion, is relevant to the field of insect ecophysiology. However, there are several concerns about the methodology and interpretation of the results that need careful consideration. I expand on these and other concerns below, and hope the author finds them useful in improving the manuscript.

Choice and justification of “control” temperature:

The decision to use 34C as the “control” requires a thorough explicit justification. Prior studies identify 33C as already stressful for this species:
• Vogt 1986 https://doi.org/10.1086/physzool.59.1.30156090
• Perl et al 2022 https://doi.org/10.3389/fevo.2021.776830
• Gerard et al 2022 https://doi.org/10.1111/gcb.16196
• Gerard et al 2023 https://doi.org/10.1242/jeb.245728
• Bretzlaff et al 2024 https://doi.org/10.1093/conphys/coae006

Additionally, the natal colonies in this study (as well as in the previous 2 studies from this research group, Wögler and Kurze 2025 and Laußer and Kurze 2025) were maintained at 25C, widely considered the developmental optimum. Without a rationale for elevating the “control” to 34C, it is difficult to interpret comparisons between “control” and heat stress treatments.

Several results from this study (as well as some results from the previous two studiesin the series) suggest that 34C may itself constitute thermal stress:
• Over 10% individuals showed deformities
• Steep early mortality (~50% within 20 days)
• No statistical differences in adult size or emergence time between “control” and heat treatment.

The absence of differences between control and treatments does not necessarily indicate heat has no effect, but instead it may indicate that 34C is also stressful. As for the Kaplan-Meier curves between natal colonies and “control”, they both show a steep early mortality, which is not seen in healthy bumblebees (Smeets and Duchateau, 2003, https://doi.org/10.1051/apido:2003026), please provide an explanation what is causing about 25% of natal control bees dying within 10 days even when food and water is provided ad lib.

I strongly recommend to provide an explicit justification for 34C as the controls. Also, include all morphological and developmental metrics (including deformity rates) from adults from the natal colonies, and discuss whether 34C produces stressed bees.

Internal inconsistency across related publications:

This manuscript appears to be part of a recent series using the identical experimental designs and changing only the heat stress regime:
• Wögler and Kurze, 2025 https://doi.org/10.1242/bio.061781

• Laußer and Kurze, 2025 https://doi.org/10.1098/rspb.2025.2029
However, the “control” outcomes differ across studies. For example, emergence times varying by 4 days across studies, and adult emergence rates changing from 30% to 80%. These discrepancies found in identical designs conducted within a short time need explanation. Please clarify, as it is difficult to assess robustness and repeatability.

Interpretation of “control” vs heat stress outcomes:

Several results are interpreted as “no significant effect of heat”, but the data appears more consistent with “controls” being heat stressed themselves. Authors show (in this study and the past two in the series) that body size, mass, and lipids do not differ between controls and heat treatments. However, if controls were also maintained at 34°C, this does not demonstrate absence of heat effects. With several studies already reporting heat-induced size shifts and costs, I recommend the authors to include a direct comparison between 25C and 34C within this study design as well as published literature. To clarify whether heat truly has no effect, it would be important to include the morphological traits (including deformity rates) and physiological timings of the natal colonies.
The Kaplan-Meier curves show a sharp adult mortality in "control" and heat treatments within 20 days of ~50%. This mortality pattern is biologically unexpected and warrants an explanation

Novelty and citation accuracy:

Several statements in the discussion overstate the novelty or mischaracterize cited work. Research over the past decade has examined developmental thermal stress and adult performance in Bumblebees. Some examples:
• Perl et al 2022 https://doi.org/10.3389/fevo.2021.776830
• Gerard et al 2022 https://doi.org/10.1093/beheco/arac045
• Gerard et al 2023 https://doi.org/10.1242/jeb.245728
• Bretzlaff et al 2024 https://doi.org/10.1093/conphys/coae006

The manuscript would benefit from reframing its contribution more precisely, maybe specifying the type of stress. For instance, the claim in L296 as “first evidence that thermal stress during pupal development leads to deferred mortality at the adult life-stage in bumblebees” when there are several published works on this topic, including this same research group’s past work.
Additionally, several references in the introduction and discussion section appear to be cited in ways that misrepresent the past literature conclusions (e.g. Reference 28 in L47, and their own reference 30 in L56, and claims in L333, and L348). I highly encourage a thorough review of each reference cited, with particular attention when past studies used controls of 25C, and their stress treatment was 33C, and how those results tie in with this study, as this is not addressed in the discussion section.

Version 1:

Reviewer comments:

Reviewer #1

(Remarks to the Author)
Major claims of the paper: Cyclic thermal stress experienced during development causes developmental damage that persists throughout adulthood.

The conclusions of this paper are novel, although similar to other papers submitted by this group; the addition of looking at thermal stress during development makes it a novel research question. I think that the authors' approach to looking at high temperature stress during an important developmental and transitional life stage shows how changing climate will impact bumblebees. The novelty of measuring how damage persists into adulthood is an important component of this paper and is often overlooked. The authors carefully considered all edits and made all adjustments satisfactorily.

Open Access This Peer Review File is licensed under a Creative Commons Attribution 4.0 International License, which permits use, sharing, adaptation, distribution and reproduction in any medium or format, as long as you give appropriate credit to the original author(s) and the source, provide a link to the Creative Commons license, and indicate if changes were made.
In cases where reviewers are anonymous, credit should be given to 'Anonymous Referee' and the source.
The images or other third party material in this Peer Review File are included in the article's Creative Commons license, unless indicated otherwise in a credit line to the material. If material is not included in the article's Creative Commons license and your intended use is not permitted by statutory regulation or exceeds the permitted use, you will need to obtain permission directly from the copyright holder.
To view a copy of this license, visit https://creativecommons.org/licenses/by/4.0/

Response to Reviewer #1:

Reviewer 1:
This manuscript demonstrates both lethal and sub-lethal effects resulting from thermal stress experienced during development. This study is well done and addresses a gap in the understanding of how global climate change impacts bumblebees by looking at how temperatures experienced during development impact adults. This manuscript was very well written, and I do not have many edits. The experiments were well-designed, and the figures represent the results well. I have included some of my recommendations to improve the manuscript below.
Response: Thank you very much for your suggests and comments, which helped us improving the quality of our manuscript.

Reviewer 1:
Introduction:
Lines 33-34: include examples of sublethal effects that have previously been found
Response: We added the following examples in lines 34-36: "…, most studies have focused on acute effects in adults, such as mortality at critical thermal maxima, metabolic disruption, and impaired flight performance and cognition [3-5]."

Reviewer 1:
Lines 48-50: include information about how adults regulate temperature and how the larvae/pupae experience different temperatures than the adults.
Response: Thanks for this suggestion. We made following changes in lines 50-55 "As behavioural responses vary widely between colonies, it is difficult to disentangle indirect effects arising from worker thermoregulation [28, 29], from direct physiological effects [30]. For example, if workers need to spend more time fanning to cool the nest or incubating the brood to maintain optimal brood temperatures (generally above ambient nest temperatures) rather than foraging and nursing [28, 29], this can affect larval development and ultimately colony fitness."

Reviewer 1:
Line 58: Does "a reduction in adult emergence" mean decreased survival? This phrasing is confusing
Response: We rephrased the sentence for greater clarity in lines 63-65: "A constant *in vitro* rearing temperature of 38°C during L4 larval and pupal development was identified as a critical thermal threshold, resulting in an increased direct mortality and higher a incidence of wing deformations [30, 31]. "

Reviewer 1:
Line 58: change "ab" to "an"
Response: Changed as suggested.

Reviewer 1:
Line 64-65: Change the phrasing of "carry-over risk" to something like residual risk, cumulative

risk, or carry-over effects
Response: Changed as suggested to “carry-over effects”.

Reviewer 1:
Methods:

Lines 108-111: How long are the bees exposed to the temperature treatments? Did they experience 1 round of the cyclic thermal regime? Or throughout development? If so, how long were the bees exposed (in days). Please clarify.
Response: Thank you for pointing this out. We added this important information in lines 119-122: “The two cyclic thermal stress treatment groups experienced a 12:12 h temperature cycle between a baseline of 34°C (night phase) and a thermal maximum (day phase) of either 37°C (37°C cyclic) or 38°C (38°C cyclic) until adult emergence (i.e. 9-10 days for males, 8-9 days for workers; Fig. 1*c*).”

Reviewer 1:
Line 137: change “contacted” to “connected”
Response: Changed to “conducted”

Reviewer 1:
Lines 137-141: clarify that you are working with adult bees. Also, it is to be assumed that these are adult bees that emerged from the pupal treatments? If that is the case please clarify here.
Response: Thank you! We specified that we conducted the morphological and physiological analyses on adult bees from individuals experiencing different thermal scenarios as pupae.

Reviewer 1:
Lines 146-148: State here briefly why you measured dry mass twice. It is stated in the following paragraph, but a brief statement would help clarify when first mentioned.
Response: Thank you. We now describe the petroleum ether extraction method in more detail in lines 168-178 to clarify the necessity of pre- and post-extraction weighing.

Reviewer 1:
Line 149-150: include citations for the extraction method
Response: Thanks. We included 2 references.

Reviewer 1:
Line 159: change “re-drying” to “re-dried”
Response: Changed.

Reviewer 1:
Results:

Line 209: report actual p value
Response: Done as suggested.

Reviewer 1:
Line 213: Again, change the term "carry-over" for clarity, and throughout the manuscript
Response: We use carry-over effects to describe the downstream effects in adults from thermally stressed pupae.

Reviewer 1:
Line 214: does "adult survival" refer to survival to adulthood? Or is it talking about adults exposed to temperature treatments. Please clarify.
Response: For clarification, we rephrased this sentence in lines 269-270 "Cyclic thermal stress exposure during pupation resulted in reduced adult survival (longevity) for both workers …." We specify that that this does not refer to survival to adulthood (emergence rate), but rather to carry-over effects on the lifespan of the emerged adults.

Reviewer 1:
Fig. 1a change the title of the y-axis to reflect that it is a proportion
Response: We added proportion (observed across colonies) and also added the predicted proportion with 95% CI from the GLMM to reflect the variance between colonies.

Reviewer 1:
Line 264: include actual pvalue
Response: Done

Reviewer 1:
Lines 273-274: did you account for body size within relative lipid content? If so please describe how this was accomplished in your analysis.
Response: As described above, we added more details about the lipid content analysis in lines 166-176.

Reviewer 1:
Discussion:

Lines 314-327: Deformities due to temperature stress has also been seen in the solitary bee Megachile rotundata, it might be helpful to include this reference to further explain your findings. doi:10.1242/jeb.243242. note this article is about low temperature stress.
Response: Thank you for this suggestion. We have included this interesting research in lines 359-362: "Interestingly, also cold stress during pupal development, mimicking a spring cold snap, in the alfalfa leafcutting bee *Megachile rotundata* (*Hymenoptera: Api*formes: Megachilidae) has been shown to cause wing deformities and reduced flight ability [47]."

Response to Reviewer #2:

Reviewer 2:
To the authors:
The authors of "Deferred mortality: cyclic thermal stress during pupation triggers irreversible carry-over costs in a key pollinator" investigated how fluctuating thermal stress during development affects bumblebee physiology including developmental time, survival, and morphology. The study, in my opinion, is relevant to the field of insect ecophysiology. However, there are several concerns about the methodology and interpretation of the results that need careful consideration. I expand on these and other concerns below, and hope the author finds them useful in improving the manuscript.

Response: Thank you very much for your careful review and invaluable comments, which helped us to substantially improve our manuscript. We included additional statistical analyses/figures and revised our discussion to address your concerns. Please review our specific responses below.

Reviewer 2:
Choice and justification of "control" temperature:

The decision to use 34C as the "control" requires a thorough explicit justification. Prior studies identify 33C as already stressful for this species:
•Vogt 1986 https://doi.org/10.1086/physzool.59.1.30156090
•Perl et al 2022 https://doi.org/10.3389/fevo.2021.776830
•Gerard et al 2022 https://doi.org/10.1111/gcb.16196
•Gerard et al 2023 https://doi.org/10.1242/jeb.245728
•Bretzlaff et al 2024 https://doi.org/10.1093/conphys/coae006

Additionally, the natal colonies in this study (as well as in the previous 2 studies from this research group, Wögler and Kurze 2025 and Laußer and Kurze 2025) were maintained at 25C, widely considered the developmental optimum. Without a rationale for elevating the "control" to 34C, it is difficult to interpret comparisons between "control" and heat stress treatments.

Response: We agree that the cited studies show that at lower temperatures can stress bumblebee colonies. However, whether these are secondary effects (due to altered colony behaviour) remains to be answered, which was a primary motivation behind our series of *in vitro* experiments. While larval development may be affected by altered worker behaviour (e.g. spending more time fanning than foraging or feeding), our study isolates the colony-level effects from direct thermal stress on pupae.

We have added the following justification for using 34°C as our baseline. Lines 102-106: "While 34°C is at the upper range of the typical nest temperature in *B. terrestris* [19], it aligns with brood temperatures, which are typically 2°C warmer than ambient nest temperatures [28] and likely represent the optimal temperature for larval development [29]. Moreover, 34°C has been

used in previous *in vitro* rearing protocols for *B. terrestris* [30, 31, 37], making it comparable to previous *in vitro* studies."

Furthermore, we changed "34°C ctrl" to "constant 34°C" and "colony ctrl" to "colony-reared" to more neutrally describe our treatment groups.

Reviewer 2:
Several results from this study (as well as some results from the previous two studies in the series) suggest that 34C may itself constitute thermal stress:
•Over 10% individuals showed deformities
•Steep early mortality (~50% within 20 days)
•No statistical differences in adult size or emergence time between "control" and heat treatment.

The absence of differences between control and treatments does not necessarily indicate heat has no effect, but instead it may indicate that 34C is also stressful. As for the Kaplan-Meier curves between natal colonies and "control", they both show a steep early mortality, which is not seen in healthy bumblebees (Smeets and Duchateau, 2003, https://doi.org/10.1051/apido:2003026), please provide an explanation what is causing about 25% of natal control bees dying within 10 days even when food and water is provided ad lib.

I strongly recommend to provide an explicit justification for 34C as the controls. Also, include all morphological and developmental metrics (including deformity rates) from adults from the natal colonies, and discuss whether 34C produces stressed bees.

Response: We acknowledge that bees in colonies typically have longer baseline survival than in our experiment. However, we do not believe our survival pattern is caused by being inherently "stressful" for the following reasons:

(1) Brood temperatures of approximately 34°C are commonly observed in *B. terrestris* colonies (Kevan et al. 2024, *Frontiers in Bee Science*) and align with behavioural observations of fanning and incubation activities of workers (Wynants et al. 2021, *Physiological Entomology*; Weidenmüller et al. 2002, *Animal Behaviour*)
(2) Our colony-reared larvae and pupae (from colonies housed at 25 ± 1°C) showed comparable survival rates (Fig. 2c, d) and a similar mortality risk (Fig. 2e).
(3) Our previous study (Laußer & Kurze 2025, *Proc B*) showed that 36°C reduced developmental duration without significantly affecting mortality, suggesting 34°C is near the thermal optimum.

We speculate that the drop in survival is likely related to a lack of social interactions, as suggested by studies in honeybees (Wild et al. 2021, *Nature Communications*) and bumblebees (Wang et al. 2022, *Current Biology*). We have acknowledged this limitation in lines 400-405: "…there was a steep decline in adult survival during the first two weeks. We speculate that this may stem from a lack of social interactions [50]. Supporting this …."

Reviewer 2:
Internal inconsistency across related publications:

This manuscript appears to be part of a recent series using the identical experimental designs and changing only the heat stress regime:
•Wögler and Kurze, 2025 https://doi.org/10.1242/bio.061781
•Laußer and Kurze, 2025 https://doi.org/10.1098/rspb.2025.2029
However, the "control" outcomes differ across studies. For example, emergence times varying by 4 days across studies, and adult emergence rates changing from 30% to 80%. These discrepancies found in identical designs conducted within a short time need explanation. Please clarify, as it is difficult to assess robustness and repeatability.

Response: Thank you for giving us the opportunity to clarify this in lines 367-389. While the *in vitro* was identical Wögler & Kurze (2025) tested L4 larvae, not pupae. The slight differences in adult emergence rates between Laußer & Kurze (2025) and the current study can be explained with high colony and temporal variation, which we statistically account for using random effects in our models. We argue that the 80% of emergence rate in our 34°C group speaks to the robustness of this study and aligns with OECD standards for *in vitro* rearing in honeybees.

Reviewer 2:
Interpretation of "control" vs heat stress outcomes:

Several results are interpreted as "no significant effect of heat", but the data appears more consistent with "controls" being heat stressed themselves. Authors show (in this study and the past two in the series) that body size, mass, and lipids do not differ between controls and heat treatments. However, if controls were also maintained at 34°C, this does not demonstrate absence of heat effects. With several studies already reporting heat-induced size shifts and costs, I recommend the authors to include a direct comparison between 25C and 34C within this study design as well as published literature. To clarify whether heat truly has no effect, it would be important to include the morphological traits (including deformity rates) and physiological timings of the natal colonies.
The Kaplan-Meier curves show a sharp adult mortality in "control" and heat treatments within 20 days of ~50%. This mortality pattern is biologically unexpected and warrants an explanation

Response: We appreciate this constructive suggestion to include a baseline comparison with colony-reared individuals. We agree that providing these data is helpful for clarifying whether 34°C acts as a stressor *in vitro*. Consequently, we have now included data from individuals developed naturally within their natal colonies (maintained at 25 ± 1°C) in the Results and updated the Discussion accordingly.

In summary, our comparison between colony-reared and 34°C *in vitro* individuals revealed that:

(1) Morphological Deformations: There was no significant difference in the proportion of deformed wings or antennae between colony-reared individuals and those reared *in vitro* at a constant 34°C.
(2) Emergence Weight: While colony-reared individuals were slightly heavier at emergence, we attribute this to the improved food provisioning naturally available to L4 larvae in a colony setting (where workers feed larvae on demand) compared to our *in vitro* protocol. This is supported by the fact that proportional pupal weight loss did not differ significantly between these groups.
(3) Allometric Scaling: The scaling relationship between emergence weight and head width did not significantly differ between colony-reared individuals and *in vitro* reared males. While the smaller variance in *in vitro* worker size made slope comparison more complex, the 37°C group remained almost identical to the colony baseline.
(4) Lipid Content: There was no significant difference in proportional lipid content between the colony-reared and *in vitro* groups.

Regarding the mortality pattern, we have added a section to the Discussion explaining that the initial drop in survival is likely a result of the absence of social interaction rather than thermal stress, as the mortality curves for colony-reared and 34°C *in vitro* bees were statistically indistinguishable (HR = 0.95, Fig. 2c-e).

We have also refined our Conclusion to acknowledge the limitations of our model system in lines 463-470: "Although our study oversimplifies realistic thermal stress scenarios... we are convinced that these findings provide valuable insights into the consequences of thermal stress during pupal development... Species that are less heat-tolerant than *B. terrestris*... would be expected to be especially at risk."

Reviewer 2:
Novelty and citation accuracy:

Several statements in the discussion overstate the novelty or mischaracterize cited work. Research over the past decade has examined developmental thermal stress and adult performance in Bumblebees. Some examples:
•Perl et al 2022 https://doi.org/10.3389/fevo.2021.776830
•Gerard et al 2022 https://doi.org/10.1093/beheco/arac045
•Gerard et al 2023 https://doi.org/10.1242/jeb.245728
•Bretzlaff et al 2024 https://doi.org/10.1093/conphys/coae006

The manuscript would benefit from reframing its contribution more precisely, maybe specifying the type of stress. For instance, the claim in L296 as "first evidence that thermal stress during pupal development leads to deferred mortality at the adult life-stage in bumblebees" when there are several published works on this topic, including this same research group's past work. Additionally, several references in the introduction and discussion section appear to be cited in ways that misrepresent the past literature conclusions (e.g. Reference 28 in L47, and their own reference 30 in L56, and claims in L333, and L348). I highly encourage a thorough review of each reference cited, with particular attention when past studies used controls of 25C, and their

stress treatment was 33C, and how those results tie in with this study, as this is not addressed in the discussion section.

Response:
Thank you for pointing this out. We apologise for any overgeneralisation and have revised our discussion to be more nuanced. We highly value the previous work by Bretzlaff et al. and Gérard et al. However, we respectfully clarify that none of the cited studies provide empirical evidence that thermal stress specifically during pupal development leads to a carry-over effect on adult longevity in bumblebees. Furthermore, our study investigates the effects of cyclic thermal stress during development, an aspect that has not been addressed in the cited literature. We believe these additions clarify that while general thermal effects are known, the specific temporal link between stress during pupal development and adult lifespan in bumblebees is a key focus of our work. Following the journal's guidelines, we have removed all priority claims and novelty statements from the manuscript.

Response to Reviewer #1:

Major claims of the paper: Cyclic thermal stress experienced during development causes developmental damage that persists throughout adulthood.

The conclusions of this paper are novel, although similar to other papers submitted by this group; the addition of looking at thermal stress during development makes it a novel research question. I think that the authors' approach to looking at high temperature stress during an important developmental and transitional life stage shows how changing climate will impact bumblebees. The novelty of measuring how damage persists into adulthood is an important component of this paper and is often overlooked. The authors carefully considered all edits and made all adjustments satisfactorily.

Response: Thank you very much for your effort!
